# Neuropsychiatric Disturbances and Diabetes Mellitus: The Role of Oxidative Stress

**DOI:** 10.1155/2019/5698132

**Published:** 2019-07-04

**Authors:** Maria Luca, Maurizio Di Mauro, George Perry

**Affiliations:** ^1^Department of Medical, Surgical Sciences and Advanced Technologies “GF Ingrassia”, University of Catania, Italy; ^2^Department of Clinical and Experimental Medicine, Andrology and Endocrinology Unit, University of Catania, Italy; ^3^Neurosciences Institute and Department of Biology, College of Sciences, University of Texas at San Antonio, USA

Oxidative stress (OS) plays a fundamental role in the pathogenesis of diabetes mellitus, neurodegenerative disorders (i.e., Alzheimer's disease (AD)), and psychiatric disturbances (i.e., depression). Interestingly, it could represent the common molecular basis explaining the high rates of comorbidity between these disorders.

This special issue is aimed at improving the current knowledge about the role of OS in the occurrence and progression of diabetes and neuropsychiatric disorders as well as its contribution to their comorbidity.

Three papers focus on OS and microbiota. In particular, L. Dumitrescu et al. underline that a peculiar microbiota type could enhance brain inflammation and OS thus favoring the occurrence of proteinopathies such as AD and Parkinson's disease. M. Luca et al. deepen the role of dysbiosis in AD, depression, and type 2 diabetes mellitus (T2D). Moreover, H.-X. Cui et al. relate the hypoglycemic effects of berberine fumarate in diabetic rats to its modulation of intestinal microflora (i.e., increase of *Bacteroidetes* and decrease of *Lachnospiraceae*).

Several contributions explore the role of OS from a multidisciplinary point of view. More specifically, G. Z. Réus et al. highlight that depression and T2D share OS, inflammation, and metabolic disturbances as common pathophysiological mechanisms, while B. Carpita et al. discuss maternal diabetes (and the related oxidative and inflammatory condition) as a risk factor for the development of autism spectrum disorders in the offspring. On the other hand, W. Ohnon et al. report the neuroprotective effects (reduction of TNF-*α*, IL-6, and other proinflammatory molecules) exerted by the combined extract of *Ozyza sativa* and *Anethum graveolens* administered to rats with experimentally induced metabolic syndrome and stroke. C. Chen et al. demonstrate that irbesartan pretreatment protects pancreatic *β*-cells from apoptosis while improving glucose levels and insulin secretion in streptozotocin-induced acute prediabetic mice. In addition, the findings by R. Li et al. indicate that Bailcalin, both in vitro and in vivo, improves cardiomyocyte hypertrophy in T2D-induced mice via antioxidative and lipid-lowering effects.

Three articles address the imbalance between reactive oxygen species production and antioxidant defense systems characterizing metabolic syndrome and diabetes. R. Vona et al. provide an overview of the biomarkers of OS in metabolic syndrome and associated diseases and comment on the possible therapeutic implications of natural antioxidants. M. Maciejczyk et al., evaluating redox homeostasis and oxidative damage in the cerebral cortex and hypothalamus of rats under insulin resistance conditions, describe an adaptive (although ineffective in terms of neuroprotection) increase in the antioxidant defense systems. On the other hand, A. Passaro et al. report, in patients with T2D, lower levels of paraoxonase-1 and lipoprotein phospholipase A2, two enzymes that, preventing the oxidative transformation of high- and low-density lipoproteins, may reduce the atherogenic risk.

All the papers collected in this special issue contribute to the current knowledge on the role of OS in neuropsychiatric disturbances and diabetes mellitus.

